# Applicability of an Experimental Grade of Hydroxypropyl Methylcellulose Acetate Succinate as a Carrier for Formation of Solid Dispersion with Indomethacin

**DOI:** 10.3390/pharmaceutics13030353

**Published:** 2021-03-08

**Authors:** Hiroshi Ueda, Yuya Hirakawa, Hironori Tanaka, Tetsuya Miyano, Katsuji Sugita

**Affiliations:** 1 Physical Chemistry, Laboratory for Medicinal Chemistry Research, Shionogi & Co., Ltd., Osaka 561-0825, Japan; tetsuya.miyano@shionogi.co.jp (T.M.); katsuji.sugita@shionogi.co.jp (K.S.); 2 Biologics, Laboratory for Advanced Medicine Research, Shionogi & Co., Ltd., Osaka 561-0825, Japan; yuya.hirakawa@shionogi.co.jp; 3 Formulation R&D Laboratory, Formulation Design Department, Shionogi & Co., Ltd., Hyogo 660-0813, Japan; hironori.tanaka@shionogi.co.jp

**Keywords:** amorphous, crystallization, enthalpy relaxation, glass transition temperature, hydroxypropyl methylcellulose acetate succinate, indomethacin, infrared spectroscopy, principal component analysis, Raman mapping, solid dispersion

## Abstract

The transformation of a crystalline drug into an amorphous form is a promising way to enhance the oral bioavailability of poorly water-soluble drugs. Blending of a carrier, such as a hydrophilic polymer, with an amorphous drug is a widely used method to produce a solid dispersion and inhibit crystallization. This study investigates an experimental grade of hydroxypropyl methylcellulose acetate succinate, HPMCAS-MX (MX), as a solid dispersion carrier. Enhancement of thermal stability and reduction of the glass transition temperature (*Tg*) of MX compared with those of the conventional grade were evaluated through thermogravimetric analysis and differential scanning calorimetry (DSC). The formation of a homogeneous amorphous solid dispersion between MX and indomethacin was confirmed by X-ray powder diffraction analysis, DSC, and Raman mapping. It was observed that 10–30% MX did not act as an anti-plasticizer, but the utilization of >40% MX caused an increase in *Tg* and reduction of molecular mobility. This could be explained by a change in intermolecular interactions, inferred from infrared spectroscopy combined with principal component analysis. HPMCAS-MX exhibited similar performance to that of conventional-grade, HPMCAS-MG. Although HPMCAS-MX has thermal properties different from those of conventional-grade HPMCAS-MG, it retains its ability as a solid dispersion carrier.

## 1. Introduction

Dosage per oral administration is a conventional pharmaceutical form, a non-invasive route that enables patients to take medicine in a way that is familiar and convenient for both adults and children [1,2,3]. It is difficult to design an oral dosage form for peptides and macromolecules because of the lower membrane permeability based on their molecular weight [4,5,6]. Hence, drug discovery and development of small molecules for simple oral administration is important and accounted for over half of the drugs approved in 2019 [7]. In the discovery, development, and manufacture of drugs, one of the most critical issues for oral absorption is an increase in poorly water-soluble small molecules, because drugs are absorbed by penetration through gastrointestinal membrane following their dissolution into the gastrointestinal fluids [8]. To overcome this issue, the conversion of the crystal form and the design of formulations to enhance drug solubility have been widely investigated [8]. Transformation of multicomponent crystal forms, such as salt and a cocrystal, is a major approach to modifying the physicochemical properties of a drug without changing its chemical structure [9,10]. Reducing the particle size to several hundred nanometers by wet milling of the crystals can enhance oral bioavailability by improving the dissolution-permeation rate [11,12,13]. The addition of lipids, oils, and emulsifiers to the aqueous solution forms an emulsion, which can solubilize the drug, and a self-emulsifying drug delivery system containing the drug and a lipid/oil/emulsifier has been investigated as a solid dosage form for poorly water-soluble drugs [14,15].

The amorphization of a crystalline drug, with disruption of the crystal lattice, is also a promising approach to enhance drug solubility and oral absorption, owing to the higher energy state [16,17]. The higher energy associated with the non-equilibrium state of the amorphous form results in molecular mobility, and crystallization is induced by physical stress or storage. The distribution of an amorphous drug into a carrier, especially a hydrophilic polymer, at the molecular level is a common strategy for designing stable amorphous formulations, which are well known as solid dispersions [16,17]. A hydrophilic polymer with a higher glass transition temperature undergoes an intermolecular interaction with the amorphous drug in such a solid dispersion, which can enhance the dissolution rate and the solubility up to a supersaturated concentration and stabilize the amorphous state through the reduction in molecular mobility. Analyses such as Fourier-transform infrared spectroscopy (FT-IR), Raman spectroscopy and solid-state ^13^C-nuclear magnetic resonance spectroscopy have revealed intermolecular interactions between amorphous drugs and polymers in solid dispersions [18,19,20,21,22]. The glass transition temperature of an amorphous drug is a vital parameter that is correlated with molecular mobility, and the miscibility of the drug and the polymer is indicated by a higher single glass transition event compared with that of the pure drug in thermal analysis [23,24,25]. The reduction in the molecular mobility of an amorphous drug through the increase in the glass transition temperature and intermolecular interactions with the polymer can inhibit crystallization [18,19,20,21,22,23,24,25].

The preparation of solid dispersions has been achieved by various methods such as evaporation of a solution, ball milling, spray drying of a solution and hot-melt extrusion (HME) [17,26]. Since environmental concerns have increased recently, HME may be employed more frequently as a solvent-free manufacturing process [27]. Various polymers have been used as solid dispersion carriers, and among these, polyvinylpyrrolidone, polyvinyl alcohol, amino alkyl methacrylate copolymers, and their derivatives have been well studied [18,19,20,21,22,23,24,25]. Cellulosic polymers used widely in pharmaceutical formulations [28], are also commonly used as solid dispersion carriers [29]. It has been reported that the enhancement of bioavailability and physical stability is greater in solid dispersions based on hydroxypropyl methylcellulose acetate succinate than in those formulated using other polymers [30,31,32].

This study aimed to investigate the physicochemical properties of an experimental grade of hydroxypropyl methylcellulose acetate succinate (HPMCAS), which modified the moiety for its commercial grade [33], applied as a solid dispersion carrier. Indomethacin was selected as the model drug, and solid dispersions of various compositions were prepared by melting the mixtures. The samples were subjected to thermal and spectroscopic analyses and physical stability tests under accelerated and stress conditions [34].

## 2. Materials and Methods

### 2.1. Materials

Indomethacin (IMC) was purchased from Fujifilm Wako Pure Chemical Corporation (Osaka, Japan). A commercial grade of hydroxypropyl methylcellulose acetate succinate (HPMCAS, Shin-Etsu AQOAT^®^ [33], type MG) and its experimental grade sample (code: “MX”) were kindly gifted by Shin-Etsu Chemical Co., Ltd. (Tokyo, Japan).

### 2.2. Thermogravimetric Analysis

Thermogravimetric analysis (TGA) of MG and MX was performed using a STA7200RV instrument (Hitachi High-Tech Science Corporation, Tokyo, Japan). A total of 5–7 mg of the sample was placed in an aluminum pan. The TGA measurement was performed at 10 °C/min at up to 350 °C. Changes in weight were recorded as a function of temperature. The data were analyzed using a TA7000 standard analysis version 11.2 (Hitachi High-Tech Science Corporation, Tokyo, Japan).

### 2.3. Preparation of Mixtures of Indomethacin and Hydroxypropyl Methylcellulose Acetate Succinate

Portions of a mixture of IMC (*X* parts by weight) with MG or MX (*Y* parts) with a total weight of 200 mg were weighed into a 2 mL stemless tube, together with two 5 mm diameter tungsten balls. A correction for the weight of absorbed water present in the MG and MX was made from the TGA results. The samples were mixed using a Shake Master Neo (Bio Medical Sciences Co., Ltd., Tokyo, Japan) at 1500 rpm for 1 min. The mixture of IMC with MG (or MX) in the weight ratio *X*:*Y* is represented as IMC-MG (or -MX) (*X*:*Y*) in this study.

### 2.4. X-ray Powder Diffraction

X-ray powder diffraction (XRPD) analysis was performed to determine the crystal or amorphous phases. The sample was placed in a hole (diameter 3 mm, depth 0.1 mm) in an aluminum plate and smoothed using a spatula. The XRPD measurements were performed using a Smart Lab diffractometer (Rigaku Corporation, Tokyo, Japan) equipped with a 9 kW rotating anode using Cu Kα radiation (λ = 1.54186 Å) and a HyPix-3000 detector. The distance between the sample and detector was 331 mm, and the diffractometer was equipped with a cross-beam optic (CBO), providing a parallel beam. By using a parallel-slit collimator with 2.5° collimation and a slit of 0.05 mm height and 0.5 mm width, the beam footprint for all measurement configurations was smaller than the width of the sample. No slit was used on the receiving side. The Cu Kα radiation point source was operated at 40 kV and 200 mA. The scan was conducted from 3° to 32° (2θ) in steps of 0.02° and a counting time of 40 s, with β axis rotation (20 rpm) during data collection. The data were analyzed using Smart Lab studio II X64 version 4.2.111.0 (Rigaku Corporation, Tokyo, Japan).

### 2.5. Differential Scanning Calorimetry (DSC)

The heat flow profiles of IMC, MG, MX, and the mixtures were measured by differential scanning calorimetry (DSC) using a Discovery DSC (TA Instruments Japan, Tokyo, Japan). Nitrogen gas was used as a purge gas at 50 mL/min, and calibration of the instrument was carried out with an indium standard. For each sample, a 1–8 mg portion was weighed into a Tzero aluminum pan and sealed with a Tzero lid. The crystalline samples were melted by heating to 172 °C at 20 °C/min, followed by cooling to −20 °C at 50 °C/min. The samples were subsequently subjected to a second heating to 172 °C at 20 °C/min to investigate the thermal behavior of the amorphous form. The data were analyzed using Trios software (version 3.3.1; TA Instruments Japan, Tokyo, Japan). The midpoint of the glass transition temperature (*Tg*) and the change in heat capacity (Δ*Cp*) were obtained from the second heating profile.

### 2.6. Calculation of Theoretical Glass Transition Temperature

The theoretical values of *Tg* for the mixtures of IMC and MG or MX were calculated by use of the Gordon-Taylor equation [19,23] (1).

*Tg* = (*w*_1_·*Tg*_1_ + *K*⋅*w*_2_⋅*Tg*_2_)/(*w*_1_ + *K*·*w*_2_) (1)

where *w*_1_ and *w*_2_, and *Tg*_1_ and *Tg*_2_ are the weight fraction and glass transition temperature (*K*) of components 1 and 2, respectively, and *Tg* is the theoretical glass transition temperature (*K*). *K* is obtained from Equation (2).

*K* = *Tg*_1_·*ρ*_1_/*Tg*_2_·*ρ*_2_ (2)

where *ρ*_1_ and *ρ*_2_ the densities of the respective components.

### 2.7. Isothermal Enthalpy Relaxation

The enthalpy relaxations of IMC and the solid dispersion containing 10–40% of MG or 10–40% of MX were studied to understand their molecular mobility according to the literature [19,23]. The amorphous IMC and the solid dispersions prepared by heating to 172 °C in the DSC measurement were aged at 30 °C for 0.5, 1, 2, 4, and 7 h. The aged samples were cooled to −20 °C at 50 °C/min and then heated to 172 °C at 20 °C/min, and the endothermic peak corresponding to the enthalpy recovery at the glass transition was analyzed. The maximum enthalpy recovery was calculated using Equation (3).

ΔH*_∞_* = Δ*Cp*(*Tg* − *T*) (3)

where Δ*H_∞_*, Δ*Cp*, *Tg*, and *T* are the maximum enthalpy recovery (J/g), the change in the heat capacity (J/g ⋅ °C) during the glass transition, the glass transition temperature before aging (°C), and the aging temperature (°C), respectively. The relaxation fraction *φ(t)* at each aging time was calculated by use of Equation (4):

*φ(t)* = 1 − (Δ*H*/Δ*H_∞_*) (4)

where Δ*H* is the observed enthalpy recovery (J/g) at each aging time during the glass transition in the DSC profile. The overall average relaxation time was calculated from the fitting of the relaxation fraction to the Kohlrausch–Williams–Watts (KWW) Equation (5):

*φ(t)* = exp (−(*t*/*τ*)*^β^*) (5)

where *t, τ*, and *β* are the aging time (h), relaxation time (h), and relaxation distribution exponent, respectively.

### 2.8. Preparation of Amorphous Indomethacin and Solid Dispersions

Amorphous IMC and the solid dispersions were prepared by melting of crystalline IMC and the IMC-MG or IMC-MX mixtures on an aluminum foil using a HS-5BH hot stirrer (AS ONE Corporation, Osaka, Japan). The melted sample was cooled to room temperature, and amorphization was confirmed by XRPD measurements.

### 2.9. Measurement of True Densities

The true densities of amorphous IMC, MG, and MX were measured using a Quantachrome Ultrapic 1200e (Anton Paar Japan K. K., Tokyo, Japan). The sample (0.9–1.5 mg) was weighed into a large cell, and the volume (cm^3^) was measured under helium gas flow. The density (g/cm^3^) was calculated from the weight and volume. The densities found for amorphous IMC, MG and MX were 1.33 g/cm^3^, 1.30 g/cm^3^ and 1.25 g/cm^3^, respectively.

### 2.10. Raman Mapping

Raman spectra of amorphous IMC, MG, and MX were obtained as reference spectra by using a RAMANtouch laser Raman microscope (Nanophoton Corporation, Osaka, Japan). The samples were placed on an aluminum plate and Raman spectroscopic measurement was performed in the carbonyl region, 1800–1500 cm^−1^, under the following conditions: excitation wavelength 785.16 nm, excitation power 123.10 mW, ND filter 100%, spectrograph center wavelength 1400 cm^−1^, grating 600 gr/mm, slit width 50 µm, exposure time 3 s, averaging 1, gain high, readout port low noise, readout speed 2 MHz, CCD temperature −70 °C, objective lens 50×/NA 0.8. The wavenumber was calibrated using a spectrum of a silicon provided with the equipment. The peak positions in the Raman spectra were assigned following a smoothing process based on the fast Fourier-transform (FFT) method, using the ACD/Spectrus Processor 2019.2.2 software (Advanced Chemistry Development Inc., Toronto, ON, Canada).

The distribution of the solid dispersions of IMC-MG (60:40) and IMC-MX (60:40) was investigated by Raman mapping based on the conditions described above. An area of 20 µm × 20 µm of the solid dispersions was measured with a resolution of 600 nm/spot under the following conditions: measurement mode, XY mapping; process, cosmic ray remover; method, median and standard deviation; area, 2; threshold, 3 × standard deviation. The spectra obtained were analyzed, and the Raman image was drawn by the area ratio between 1680.1 ± 10.7 cm^−1^ and 1740.0 ± 10.6 cm^−1^ using a Raman Imager 2 (Nanophoton Corporation, Osaka, Japan).

### 2.11. Fourier-Transform Infrared Spectroscopy

The molecular states of amorphous IMC, MG, MX, and the solid dispersions were investigated by FT-IR using a VERTEX 70 spectrometer (Bruker Optics K.K., Tokyo, Japan). The number of the scan time and resolution were 64 and 4 cm^−1^, respectively. The measurements focused on a wavenumber range in the carbonyl region, 1800–1500 cm^−1^. The peak heights in the infrared spectra of the samples were normalized by a Min/Max procedure using OpusLab version 5.0 (Bruker Optik GmbH, Milton, ON, Canada). The peak positions were assigned using the ACD/Spectrus Processor 2019.2.2 software (Advanced Chemistry Development Inc., Toronto, ON, Canada).

### 2.12. Multivariate Analysis of Infrared Spectra

The FT-IR spectra were analyzed by multivariate analysis. The principal component analysis (PCA) was performed by using an Unscrambler X, version 10.5 (CAMO software, Oslo, Norway), wherein the FT-IR spectral peak height was utilized as a function of wavenumber as the input parameter. Full cross-validation was applied and singular value decomposition was used as an algorithm. The PCA score plot was prepared using PC-1 and PC-2. The loading plot as a function of wavenumber was also used to interpret the PCA score plot based on PC-1 and PC-2 in accordance with the literature [35].

### 2.13. Isothermal Crystallization

The crystallization tendencies of amorphous IMC and the solid dispersions were investigated. The samples, occupying a circular depression (diameter 3 mm, depth 0.1 mm) in an aluminum plate, were placed in two desiccators containing silica gel. The desiccators were stored at 40 and 60 °C under accelerated and stress conditions, respectively [34]. The aluminum plates were subjected to XRPD measurements 1, 3, 7 and 28 days after storage.

## 3. Results and Discussion

### 3.1. Structure of Hydroxypropyl Methylcellulose Acetate Succinate

Hydroxypropyl methylcellulose acetate succinate (HPMCAS) is a pharmaceutical cellulosic polymer that incorporates functional groups such as methoxy, hydroxypropoxy, acetyl, and succinyl groups [33]. Three grades of HPMCAS were supplied by Shin-etsu Chemical Co., Ltd. with different weight ratios of the functional groups described above, in which an increase in the methoxy group, hydroxypropoxy group, or acetyl group, and a decrease in the succinyl group, increases the pH required for solubilization of HPMCAS [33]. The lower (LG/LF), middle (MG/MF), and higher (HG/HF) grades of HPMCAS were solubilized into aqueous solutions at pH ≥ 5.5, ≥6.0, and ≥6.5, respectively. An experimental grade of HPMCAS (MX) was designed for HME application, based on the middle grade for the change in its thermal properties.

### 3.2. Thermal Stability of Hydroxypropyl Methylcellulose Acetate Succinate

**Figure 1:**
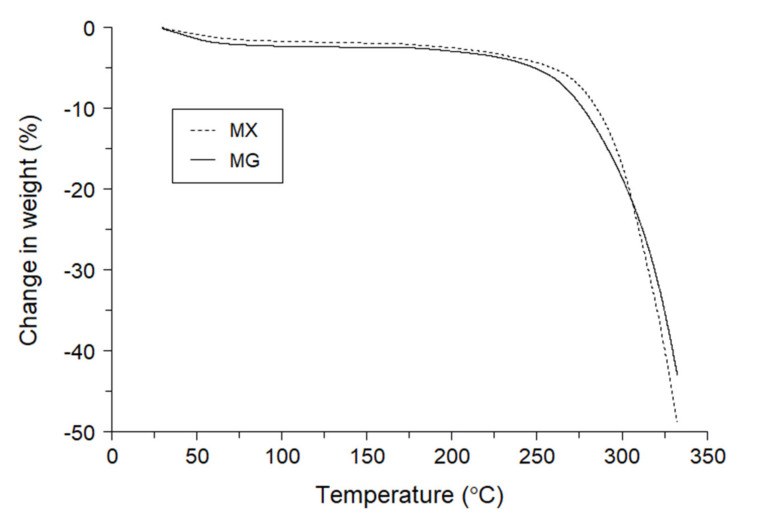
Thermogravimetric profiles of hydroxypropyl methylcellulose acetate succinate (HPMCAS)-MG (MG) and HPMCAS-MX (MX).

The thermal stabilities of MG and MX were investigated. Figure 1 shows the thermogravimetric profiles of MG and MX obtained by TGA measurements at temperatures up to 350 °C. The profiles showed weight reductions corresponding to 2.1 ± 0.1% and 1.6 ± 0.0%, respectively up to 80 °C, suggesting desorption of water by heating. Both samples maintained their weights with little further change up to 200 °C. Above 250 °C, a rapid weight reduction, reflecting thermal degradation, was observed for MG, with an onset temperature of 277.7 ± 3.4 °C. A similar profile was also observed for MX, but the onset of thermal degradation occurred at 288.6 ± 2.6 °C. These results suggest that the thermal stability of MX is higher than that of MG.

### 3.3. Glass Transition Temperature of Indomethacin and Solid Dispersions

**Figure 2:**
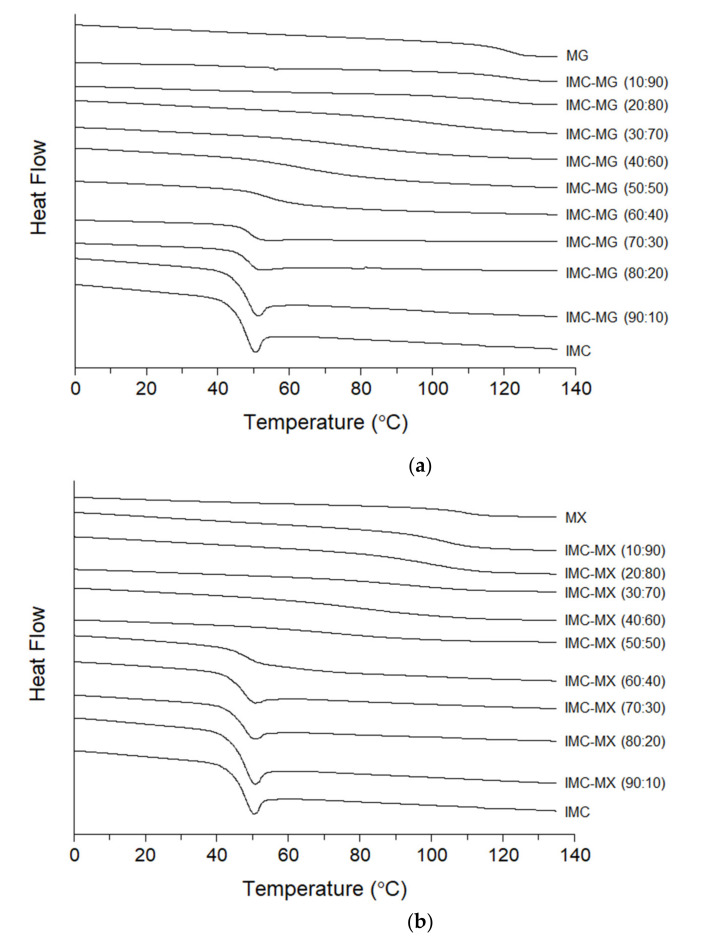
Heat flow profiles on the second heating following melting of indomethacin (IMC), HPMCAS-MG (MG)/HPMCAS-MX (MX) and the solid dispersions: (**a**) IMC-MG and (**b**) IMC-MX.

To investigate the thermal properties, the heat flow profiles of IMC, MG, MX, and the IMC-MG and -MX mixtures were measured by DSC. Figure 2a,b show the heat flow profiles of IMC-MG and IMC-MX, respectively, during the second heating process following the first heating and cooling. None of the samples showed crystallization behavior during the cooling process, suggesting the formation of an undercooled amorphous glass. Glass transition behavior was observed for IMC and MG at 48.3 ± 0.2 °C and 121.3 ± 1.2 °C, respectively (Figure 2a). These were similar results to previously reported values [18]. The IMC-MG mixtures showed a single *Tg* in all the compositions, which rose from 48.7 ± 0.5 °C to 120.3 ± 1.5 °C as the weight ratio of MG increased from 10% to 90%. Similar profiles were observed for the IMC-MX mixtures. Figure 2b shows the heat flow profiles of IMC, MX, and IMC-MX mixtures. The *Tg* of MX was 107.9 ± 0.4 °C which was lower than that of MG despite the higher thermal stability of MX (Figure 1). The IMC-MX mixtures also showed a single *Tg* in all compositions, with reduction depending on the weight ratio of IMC. These results suggest that, after melting, IMC formed a miscible solid dispersion with both MG and MX.

**Figure 3:**
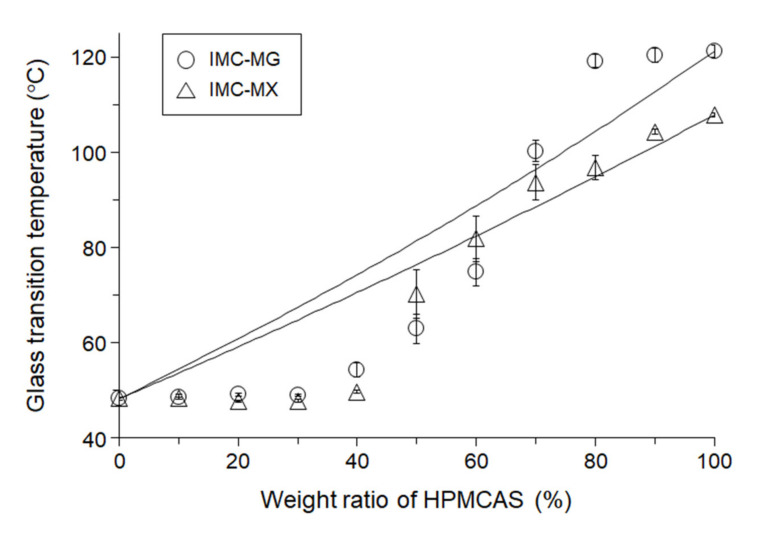
Glass transition temperatures (*Tg*s) of indomethacin (IMC), HPMCAS-MG (MG)/HPMCAS-MX (MX) and the solid dispersions prepared by the melting on the first heating by differential scanning calorimetry. The error bars represent standard deviation of *n* = 3. The lines represent the theoretical *Tg*s calculated from Gordon-Taylor equation.

Figure 3 shows the *Tg* values as a function of composition, with the solid lines the theoretical *Tg* values calculated from the Gordon–Taylor equation. The addition of 10–30% of MG did not have an anti-plasticizing effect on the *Tg* of IMC. The *Tg* of IMC-MG increased with over 40% of MG and the *Tg* values of IMC-MG (60:40), IMC-MG (50:50), and IMC-MG (40:60) were 54.3 ± 1.5 °C, 63.0 ± 3.1 °C, and 74.8 ± 2.8 °C, respectively, which are significant negative deviations from the theoretical values. On the other hand, the *Tg*s of IMC-MG (30:70), IMC-MG (20:80), and IMC-MG (10:90) were 100.3 ± 2.3 °C, 119.2 ± 1.4 °C and 120.3 ± 1.5 °C, respectively, with positive deviations from the theoretical values. A similar tendency has been reported in other studies, in which lower concentrations of HPMCAS did not show anti-plasticizing effects on carbamazepine, felodipine, and indomethacin [21,22,24]. IMC-MX showed a similar variation in glass transition temperature with composition. The *Tg* of IMC-MX (90:10) was 48.3 ± 0.1 °C, which is comparable to that of IMC. Addition of 20% and 30% of MX resulted in small reductions in *Tg* from 48.7 ± 0.5 °C to 47.7 ± 0.1 °C and 47.8 ± 0.3 °C, respectively. 40% MX acted as an anti-plasticizer for IMC, producing a *Tg* of 49.7 ± 0.3 °C. Increasing the amount of MX significantly elevated *Tg* of the solid dispersion to 70.3 ± 5.1 °C, 81.9 ± 4.8 °C, 93.7 ± 3.8 °C, 96.9 ± 2.5 °C and 104.3 ± 0.5 °C for 50%, 60%, 70%, 80% and 90% of MX, respectively. These results suggest that a lower amount of MG/MX, 10–30%, does not act as an anti-plasticizer for IMC, but a change in the molecular state of the solid dispersion at a 60:40 ratio of MC to MG/MX resulted in elevation of *Tg*.

### 3.4. Molecular Mobility of Indomethacin and Solid Dispersions

**Figure 4:**
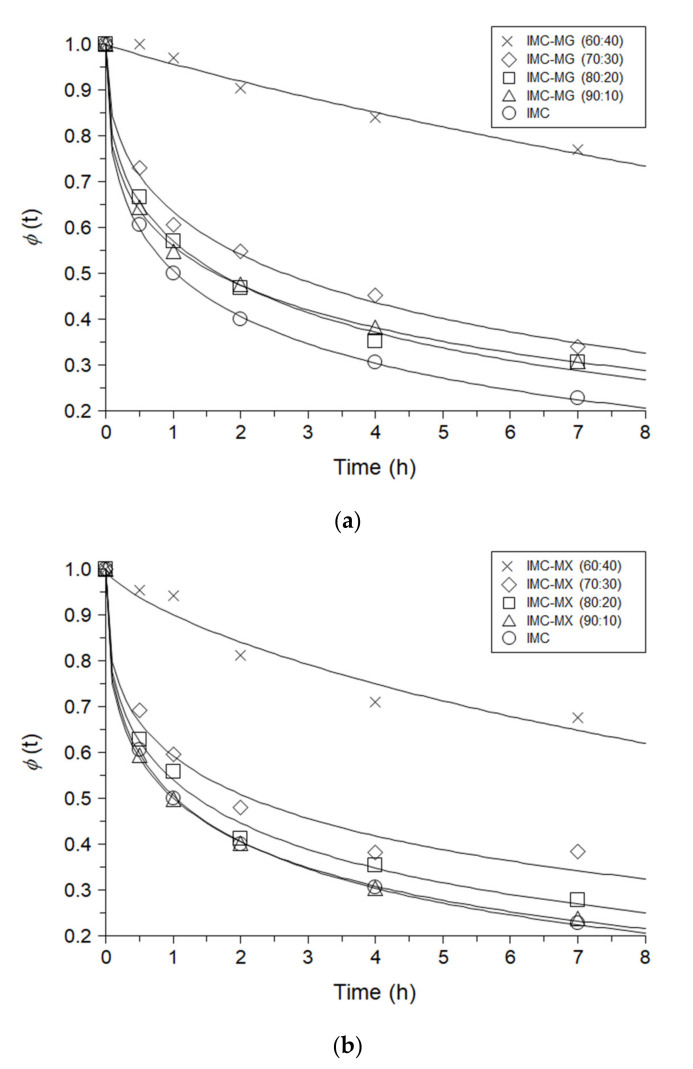
Enthalpy relaxation profiles of indomethacin (IMC), and the solid dispersions comprising of (**a**) IMC-HPMCAS-MG (MG) and (**b**) IMC-HPMCAS-MX (MX) aged at 30 °C. The lines represent the theoretical values by fitting to the Kohlrausch–Williams–Watts (KWW) equation.

To investigate the molecular mobility, the enthalpy relaxation of the solid dispersions containing 10–40% MG/MX as well as IMC were studied. Figure 4a,b show the enthalpy relaxation profiles of IMC-MG and IMC-MX with aging at 30 °C, respectively. IMC shows the reduction of the relaxation fraction depending on the aging time. Addition of 10–30% of MG/MX delayed the relaxation profile depending on the polymer ratio, but not significantly. The reduction of the enthalpy relaxation factions of IMC-MG (60:40) and IMC-MX (60:40) significantly delayed. Table 1 shows the KWW parameters *τ*, *β* and *τ^β^* of IMC and the solid dispersions containing 10–40% MG/MX. Since it was reported that *τ^β^* was not relatively affected by the aging time [36], this parameter was used for comparison of the relaxation rate among the samples. *τ^β^* of IMC was 1.47 h which was changed by addition of 10% MG to 1.73 h, but that of IMC-MX (90:10) was 1.63 h. Increase in ratio of MG/MX gave higher *τ^β^* which were 1.79, 2.20, 1.45 and 1.91 h at 10% MG, 20% MG, 10% MX and 20% MX, respectively. Remarkable change was observed in IMC-MG (60:40) and IMC-MX (60:40) of which *τ^β^* were 23.08 and 9.63 h, suggesting that the molecular mobility of the solid dispersion significantly decreased at 60:40 ratio of IMC to MG/MX. The results of the TGA and the DSC suggest that MX has thermal properties of lower *Tg* and higher thermal stability than those of MG but solid dispersion prepared from those polymers gave similar tendencies.

### 3.5. Distribution Images of Indomethacin and Hydroxypropyl Methylcellulose Acetate Succinate in Solid Dispersion

**Figure 5:**
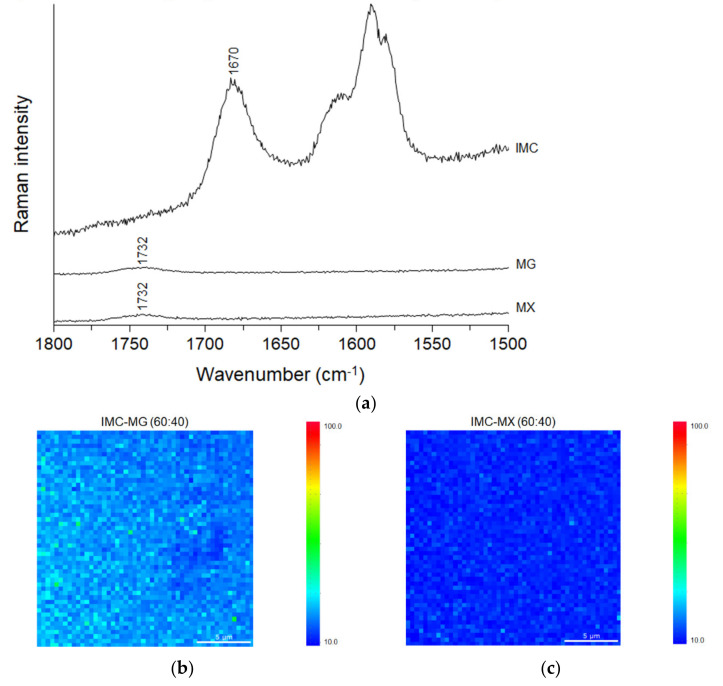
(**a**) Raman spectra of amorphous indomethacin (IMC), HPMCAS-MG (MG) and HPMCAS-MX (MX), and Raman images of the solid dispersions of (**b**) IMC-MG (60:40) and (**c**) IMC-MX (60:40).

The distributions of IMC and MG/MX in the solid dispersion were evaluated using Raman mapping. Figure 5a shows the Raman spectra of amorphous IMC, MG, and MX. The spectrum of IMC was consistent with the reported pattern of amorphous IMC [37], and the amide C=O stretching vibration appeared at 1670 cm^−1^. Both the Raman spectra of MG and MX showed a small broadened peak at 1732 cm^−1^, which could be distinguished from the peak of IMC. The distributional Raman images of IMC and MG/MX in the solid dispersion containing 40% MG/MX were drawn based on the ratio of the peaks at 1670 cm^−1^ to 1732 cm^−1^. Figure 5b,c show the Raman images of IMC-MG (60:40) and IMC-MX (60:40), respectively, at 20 µm × 20 µm with a resolution of 600 nm. Both samples gave homogeneous distribution images of IMC and MG/MX in the solid dispersion, which agreed with the single *Tg* obtained from the DSC profile (Figure 2a,b).

### 3.6. Intermolecular Interactions between Indomethacin and Hydroxypropyl Methylcellulose Acetate Succinate in Solid Dispersions

**Figure 6:**
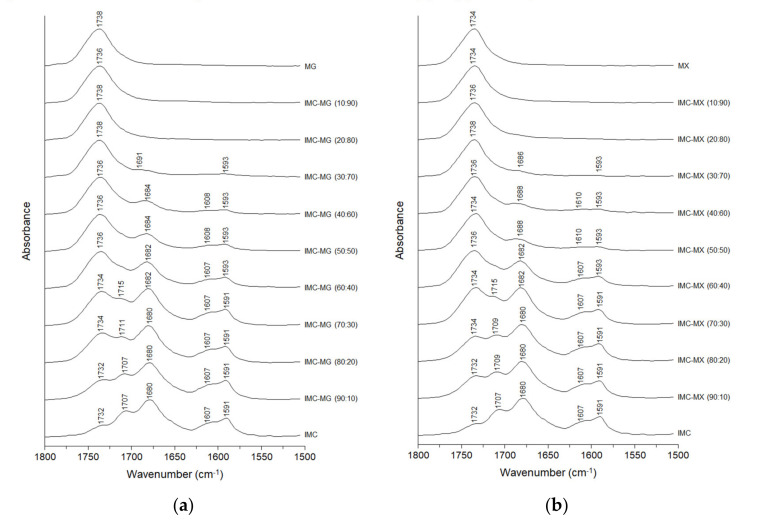
Fourier-transform infrared spectra of amorphous indomethacin (IMC), HPMCAS-MG (MG), HPMCAS-MX (MX), and the solid dispersions: (**a**) IMC-MG and (**b**) IMC-MX.

Intermolecular interactions between IMC and MG/MX in the solid dispersions were investigated by FT-IR analysis. Figure 6a shows the FT-IR spectra of amorphous IMC, MG, and the solid dispersions containing 10–90% of MG. The peaks of IMC appeared in the range 1800–1650 cm^−1^ and showed shifts and changes in shape depending on the weight ratio of MG, although peaks in the range 1650–1500 cm^−1^ were not affected. The peaks of IMC at 1732 cm^−1^, 1707 cm^−1^, and 1680 cm^−1^ were assigned to the C=O stretch of the free acid, the cyclic dimer, and the amide C=O, respectively [18]. The free acid peak shifted from 1732 cm^−1^ to a relative higher wavenumber on the addition of MG although the shift was only 2–4 cm^−1^ in the range of 10–70% of MG. The peak assigned to the carbonyl C=O of the cyclic dimer showed a shift to higher wavenumber, from 1707 to 1715 cm^−1^ in IMC-MG (70:30). In addition, this peak almost disappeared in IMC-MG (60:40), suggesting disruption of the cyclic dimer of IMC-IMC and formation of intermolecular interactions with MG. It is possible that the amide C=O also formed an intermolecular interaction with MG, because a peak shift to higher wavenumber was observed in the solid dispersions containing over 30% of MG. Figure 6b shows the FT-IR spectra of IMC, MX, and the solid dispersions containing 10–90% MX. The solid dispersions of IMC-MX gave FT-IR spectra and changes in the patterns in the solid dispersions quite similar to those of IMC-MG, with the peak assigned to the cyclic dimer almost disappearing at the 60:40 ratio of IMC to MX. From these results, it can be concluded that MG and MX were in similar molecular states in the solid dispersions with IMC. A similar result was reported for the solid dispersion of IMC and hydroxypropyl methylcellulose [18].

**Figure 7:**
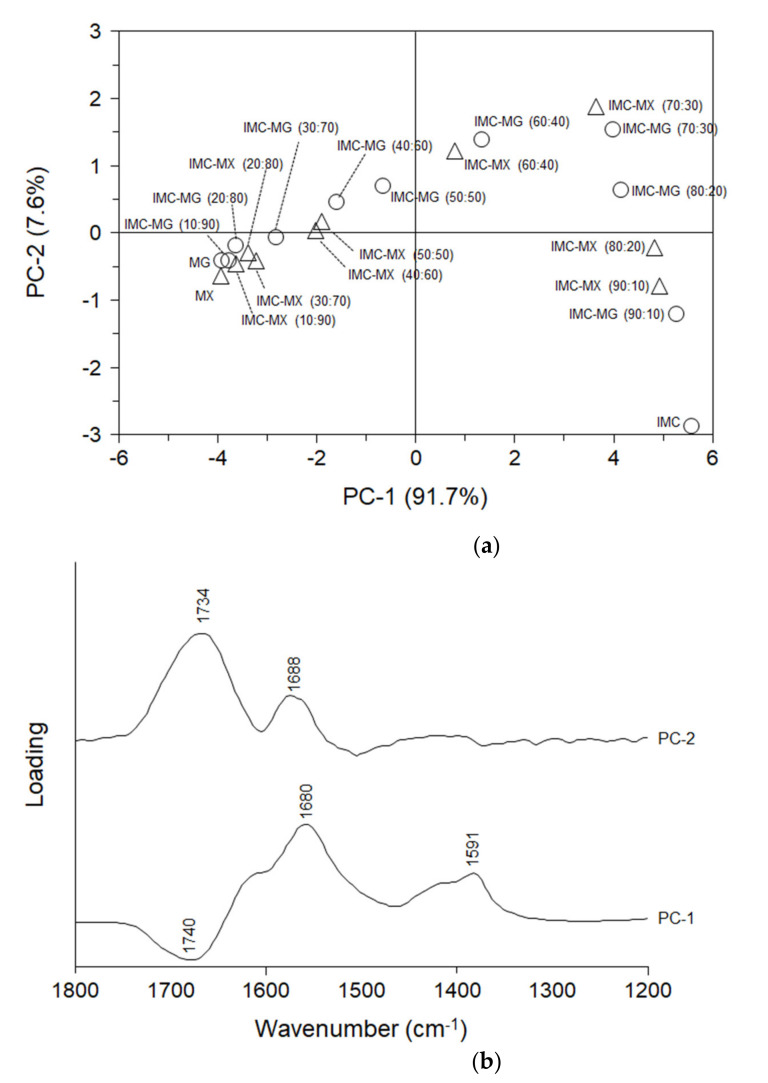
(**a**) Principal component analysis (PCA) score plot and (**b**) principal component (PC) loadings of PC-1 and PC-2 for the infrared spectra of amorphous indomethacin, HPMCAS-MG/HPMCAS-MX and the solid dispersions.

To understand the change in the FT-IR spectra of the solid dispersion, a multivariate analysis was performed. Figure 7a shows the PCA score plot. The PCA score created was explained by PC-1 (91.7%) and PC-2 (7.8%) in the calibration. The validation process yielded similar values: PC-1 (90.3%) and PC-2 (8.9%). In both the calibration and validation, approximately 99% of the PCA score model could be explained by PC-1 and PC-2 [35]. IMC was plotted in the lower right quadrant, which showed an upward shift with the addition of MG/MX up to IMC-MX (70:30) and IMC-MG (70:30). However, further addition of MG/MX shifted the plots toward the lower left, depending on the weight ratio of MG/MX. The plots of the solid dispersions containing 70% and 90% MG/MX appeared in the lower left quadrant, close to the plots of MG and MX. Figure 7b shows the PC loadings of PC-1 and PC-2. Comparing the PC-1 loading with the FT-IR spectra (Figure 6a,b), it can be seen that the positive parts of PC-1 appear at 1680 cm^−1^ and 1591 cm^−1^, which reflects the spectrum of IMC, but the negative part at 1740 cm^−1^ almost agrees with the spectrum of MG/MX. The positive part at 1688 cm^−1^ in PC-2 can also be related to a peak of IMC, as in the case of PC-1. On the other hand, the peak of MG/MX at 1734 cm^−1^ appeared as a positive maximum at 1734 cm^−1^ in PC-2, although it appeared negative in PC-1. Based on these results, the PCA score plot can be interpreted. PC-1 almost reflects the content of IMC in the solid dispersion, inducing a shift of the plot toward the left with a reduction in the weight ratio of IMC. PC-2 reflects mainly the peak of MG/MX, but partially that of IMC. The upward shift of the plot from IMC to IMC-MG (70:30)/IMC-MX (70:30) was induced by an increase in the weight ratio of MG/MX, whereas the downward shift of the plot in the solid dispersion containing 40–90% MG/MX must reflect the reduction of the peak of IMC at around 1680–1690 cm^−1^, since this peak was included in PC-2. The PCA score plot clearly shows a remarkable change in the shifts at a 60:40 ratio of IMC to MG/MX, the composition that corresponds with an increase in *Tg* and a reduction in molecular mobility (Figure 3 and Figure 4a,b). The thermal and spectroscopic analyses reveal that both MG and MX formed relatively strong intermolecular interactions with IMC at weight ratios of 40% or more, but not at 10–30%.

### 3.7. Crystallization Tendency of Indomethacin and Solid Dispersions

**Figure 8:**
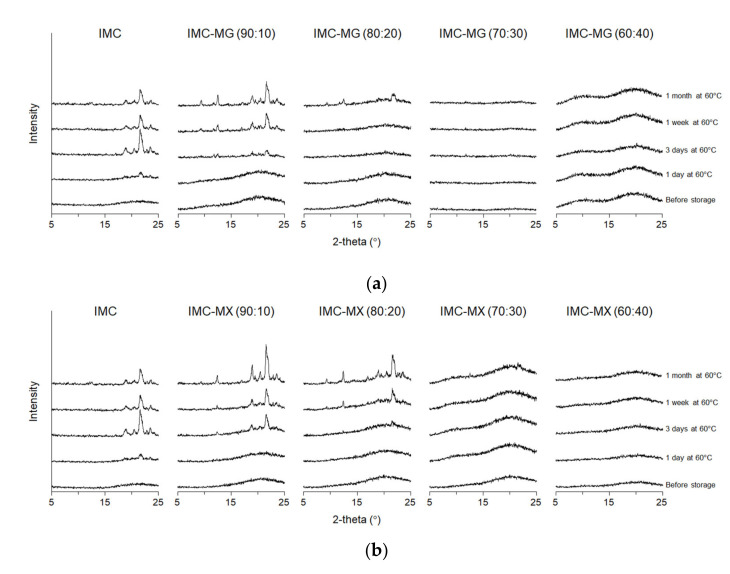
X-ray diffraction patterns of amorphous indomethacin (IMC) and the solid dispersions with 10–40% of indomethacin (IMC), HPMCAS-MG (MG)/HPMCAS-MX (MX) before and after storage at 60 °C. (**a**) IMC-MG and (**b**) IMC-MX.

To maintain the amorphous form of a solid dispersion during storage is essential for the pharmaceutical dosage, and the crystallization behavior of the solid dispersion was, therefore, investigated and compared to that of amorphous IMC. Amorphous IMC and the solid dispersions were stored at 40 °C and 60 °C at which IMC and the solid dispersions containing 10–40% MG/MX were in the glass and the supercooled liquid states, respectively, whereas the 50–90% MG/MX dispersions were glass at both temperatures. All samples were stable and showed no crystallization behavior at 40 °C (data not shown). Figure 8a,b show the XRPD patterns of IMC-MG and IMC-MX stored at 60 °C. Rapid crystallization, demonstrated by an X-ray diffraction peak at around 22°, was observed 1 day after storage for IMC, and was inhibited by only 10% MG/MX. Both IMC-MG (90:10) and IMC-MX (90:10) crystallized within 3 days. Although IMC-MG (80:20) maintained its amorphous state for 7 days, IMC-MX (80:20) crystallized within 3 days. No crystallization occurred for 7 days in IMC-MG (70:30) and IMC-MX (70:30), but IMC-MX (70:30) showed the small diffraction peak. The solid dispersions containing over 40% of the polymer maintained an amorphous state during the period of study. These results could be caused by a reduction in molecular mobility (Figure 4a,b and Table 1) resulting from the formation of intermolecular interactions (Figure 6a,b and Figure 7a,b). Although a slight difference in physical stability was observed between the samples containing 80–70% IMC, MX showed almost a similar ability to that of MG as a solid dispersion carrier.

## 4. Conclusions

This study investigated the physicochemical properties of an experimental grade of HPMCAS, “HPMCAS-MX”, designed by modification of the functional group of MG/MF grade. An increase in the thermal degradation temperature of MX compared with that of MG was suggested by TGA measurements. MX had a *Tg* approximately 13 °C lower than that of MG and formed miscible solid dispersions with IMC after melting. Lower amounts of MX corresponding to 10–30% did not elevate the *Tg* and reduce the molecular mobility of IMC. Addition of over 40% of MX increased the *Tg* with an accompanying reduction in the molecular mobility, which contributed to the physical stability at 60 °C. These results could be explained by the formation of relatively strong intermolecular interactions between the components, manifested by changes in the FT-IR spectra. Based on this evidence, we conclude that HPMCAS-MX can be used as a solid dispersion carrier which exhibited similar performance to the conventional HPMCAS-MG. Furthermore, it is suggested that changes in the thermal properties of MX might enable heating processes such as hot-melt extrusion to be performed at lower temperatures than those of conventional HPMCAS while maintaining the performance of the solid dispersion. We will focus on this matter in a future study.

## Figures and Tables

**Table 1:** Kohlrausch–Williams–Watts parameters obtained from the enthalpy relaxation profiles.

Sample	*τ* (h)	*β*	*τ^β^* (h)
IMC	2.60	0.40	1.47
IMC-MG			
(90:10)	4.45	0.37	1.73
(80:20)	4.11	0.41	1.79
(70:30)	6.21	0.43	2.20
(60:40)	27.79	0.94	23.08
IMC-MX			
(90:10)	3.49	0.39	1.63
(80:20)	2.63	0.38	1.45
(70:30)	5.83	0.37	1.91
(60:40)	21.97	0.73	9.63
